# Surgical management of pelvic Ewing’s sarcoma

**DOI:** 10.4103/0019-5413.69312

**Published:** 2010

**Authors:** Mayil Vahanan Natarajan, M Mohamed Sameer, Jagdish Chandra Bose, Kunal Dheep

**Affiliations:** Department of Orthopaedic Surgery and Traumatology, Madras Medical College and Government General Hospital, Chennai - 600 003, India

**Keywords:** Adjuvant chemotherapy, Ewing’s sarcoma, pelvic neoplasms, pelvic resections, reconstruction

## Abstract

**Background:**

Despite advances in adjuvant therapy, Ewing’s sarcoma of the pelvis remains an anatomic site with a poor prognosis due to its relative inaccessibility, complex anatomy, and limited reconstructive options available. This study evaluates the role of surgery in the management of patients with pelvic Ewing’s sarcoma who also have received conventional radiation therapy and chemotherapy.

**Materials and Methods:**

From July 1990 to July 2006, we received 10 patients with Ewing’s sarcoma of pelvis at our center. Nine patients were in stage II B and one in Stage III at the time of presentation to us. All patients underwent surgical resection after preoperative chemotherapy with or without radiotherapy, which was given at the discretion of the referral center. Reconstruction was attempted using plate osteosynthesis in four patients, SS wires and screws in three patients, free fibular strut graft in one patient, and none was done in two patients.

**Results:**

Functional outcome assessed by Enneking’s criteria revealed excellent outcome in two patients, good outcome in five patients, and poor outcome in two patients. At a mean followup of 10.3 years, seven patients remained free from the disease, and three patients died. The 5- and 10-year cumulative survival (Kaplan Meier method) was 63% and 34%, respectively.

**Conclusion:**

This study demonstrates that surgery plus chemotherapy and radiation therapy is helpful for treating patients with pelvic Ewing’s sarcoma, particularly in achieving local control.

## INTRODUCTION

Ewing’s sarcoma is the most common malignant bone tumor of the pelvis in children and adolescents.^1^ The inaccessibility of the tumor due to a complex pelvic anatomy as well as its deep location makes early diagnosis and management of this sarcoma a challenging task. The prognosis remains poorest in the pelvis with survival rates at 5 years of approximately 50% in patients with Ewing’s sarcoma,^2^–^4^ though advancements in multimodal treatments have improved the survival rates. Extensive pelvic surgeries are demanding for the surgeon and for the patient because of the navigational difficulty in the pelvis, numerous muscle attachments, and the proximity of the major blood vessels, nerves, and visceral organs. It is difficult to achieve local control, limb salvage, and at the same time good function. The use of neoadjuvant chemotherapy, followed by secondary local control for the primary site with surgical resection, radiation, or both, is an accepted regimen for patients with Ewing’s sarcoma. This study evaluates the role of surgery in the management of patients with pelvic Ewing’s sarcoma who also received conventional chemotherapy with or without radiotherapy.

## MATERIALS AND METHODS

**Figure 1:**
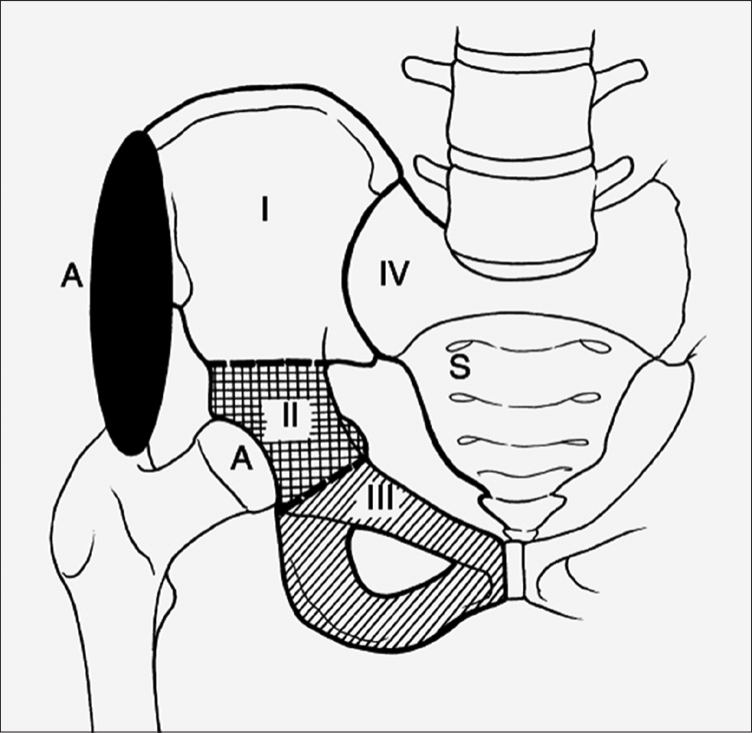
A line diagram showing types of pelvic resection

Between July 1990 to July 2006, 10 patients underwent limb-sparing pelvic resections [Figure 1] for Ewing’s sarcoma. Their mean age was 16.7 years (10-24 years). The age distribution and the diagnoses are shown in Table 1. All our patients were referred from elsewhere. Most of them had received conventional chemotherapy and radiotherapy at the time of referral. The tumors were assessed by plain radiography, CT, and MRI. Definitive diagnosis was confirmed histopathologically in all patients. Staging was done for all patients at the time of presentation using the Musculo-Skeletal Tumour Society System.^5^ Out of the 10 patients, 9 had localized tumors (stage IIB) and 1 had tumor metastasis to lung (stage III) at the time of diagnosis. All the patients we have treated were referral patients, and most of them were large tumors at presentation (more than 15 cm on clinical examination). They had undergone both chemotherapy and radiotherapy at the discretion of the referees. All patients had conventional chemotherapy pre- and postoperatively. Standard VACA regimen consisted of vincristine (VC), actinomycin (AC), cyclophosphamide (CP), and doxorubicin (AD). Few cases had additional ifosfamide (IF) alone or combined with etoposide (ET). Postoperative radiotherapy was given in patients with marginal resection. Post-therapy necrosis is a recent prognostic factor, which was not given importance in our initial cases.

**Table 1:** Clinical data of patients

Age	Sex	Part involved	Stage	Adjuvant therapy	Surgery done	Date of surgery	Oncological outcome		Oncologic status	Functional outcome
							Local recurrence	Distant metastasis		
20	M	Left para-acetabulum+ischium	II B	CT + preop RT	Type II + III resection iliofemoral fusion with plate osteosynthesis	7/1990	No	No	NED	G
24	M	Left Ilium	II B	CT + preop RT	Type I resection (no reconstruction)	1/1996	No	No	DOC	-
11	F	Right Ilium with periacetabulum	III	CT + preop RT	Type I + II resection ischiofemoral fusion with screws and ss wires	7/1997	No	Multiple secondaries	DOD	P
27	M	Right Ilium with periacetabulum	II B	CT + postop RT	Type I + II + sacral resection and ischiofemoral fusion with ss wires	9/1999	No	No	NED	E
10	M	Right Ilium with periacetabulum	II B	CT	Type I + II hemi ishio femoral fusion with ss wires	05/2000	No	Multiple secondaries	DOD	P
17	M	Right periacetabulum with ischium	II B	CT + postop RT	Type II + III + part of ilium resection and iliofemoral fusion with plate osteosynthesis and mesh repair	9/2000	No	No	NED	E
13	M	Right Ilium with periacetabulum	II B	CT	Type I + II resection and ilio femoral fusion with plate osteosynthesis	2/2001	No	No	NED	G
17	M	Right ischium	II B	CT	Type III resection (no recontruction)	3/2003	No	No	NED	G
15	M	Right ilium	II B	CT + preop RT	Type I + sacral resection and reconstruction with fibular graft and screws	2/2005	No	No	NED	G
13	F	Right ischium	II B	CT + preop RT	Type II + III resection iliofemoral fusion with plate osteosynthesis and mesh repair	7/2006	No	No	NED	G

NED: No evidence of disease at last followup; DOC: Died of complications; CT: Chemotherapy; RT: Radiotharepy

### Technique of operation

Preparation for the surgery included catheterization of the bladder to maintain an empty bladder during surgery, and bowel preparation using an enema. The surgeries were performed under general anesthesia with the patient in the lateral position, and were covered by prophylactic broad-spectrum antibiotics including metronidazole. Meticulous preoperative planning was done to achieve wide resection of the tumor in all patients using available investigations knowing very well that intraoperative decisions may vary because of the difficult anatomy and unexpected tumor invasion. Preoperative counseling about the need to eradicate the tumor was given to all the patients and were warned that it might be impossible technically to preserve the limb; they all consented to amputation in such conditions.

We used a Utilitarian pelvic incision for all the patients. Incision begins at the posterior iliac spine and extends along the iliac crest to the anterior superior iliac spine. It is separated into two arms. One is carried along the inguinal ligament up to the symphysis pubis and the other arm turns distally over the anterior thigh for one-third of the length of the thigh and then curves laterally just posterior to the shaft of the femur below the greater trochanter and follows the insertion of gluteus maximus.

**Figure 2:**
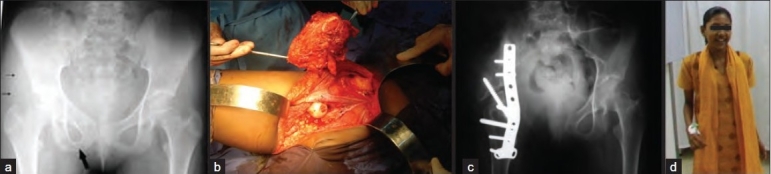
(a) Preoperative X-ray (anteroposterior view) of a patient showing Ewing’s sarcoma involving right ischium (thick black arrow) and soft tissue extent (thin black arrows). (b) Intraoperative clinical picture of same patient showing Type I+II pelvic resection. (c) Postoperative X-ray (anteroposterior view) after Type I + II resection and attempted iliofemoral fusion with plate osteosynthesis after 3 years. (d) Clinical followup after 3 years with good functional outcome

The operative details varied from patient to patient, but there were few common features. All patients underwent internal hemipelvectomy with attempted wide margins of resection [Figure 2]. No frozen section studies were used intraoperatively in this study. During the initial part of the series, the average operative time was 8 h, which was reduced to average of 4 h in the later part of the series. Similarly transfusion requirements decreased from 6-8 units to 4 units. Reconstruction procedure was attempted using conventional orthopedic implants (plates, screws, wires) or fibular strut graft as necessary, if immediate poststability of the limb was at stake. We did not use prosthesis in this subgroup of patients with pelvic tumors. The postoperative rehabilitation was tailored according to the type of resection and reconstruction and the improvement of the patient. In general, all of them were advised 8 weeks of complete bed rest and after that they were started on partial weight bearing with walking frame support. Later these were weaned and appropriate orthoses were prescribed for these patients for mobilization. All these patients had shortening (range 4-9 cm) depending upon the type of resection and reconstruction. Appropriate foot and ankle orthoses were prescribed for these patients to aid their mobility. All patients were followed up at regular intervals - monthly followup in first year, once in 2 months in second year, and 6 monthly thereafter. Recurrences were defined as local, systemic, or both. Survival rates were analyzed by Kaplan Meier Estimation method.^6^

## RESULTS

The ilium was the commonest site involved (n=6) followed by ischium. Six patients had tumor involving more than one pelvic area. One patient had multiple secondaries in the chest at the time of presentation. At the time of review, seven patients were alive and free from local and distant metastatic disease, at a mean followup of 10.3 years (range 3 to 19 years). One patient had severe intraoperative bleeding and succumbed to complications of massive transfusion in immediate postoperative period. Two patients died of metastasis disease - one had metastasis at the time of presentation and other developed a large tumor diagnosed to have metastasis at the immediate followup period.

Major intraoperative complications included tumor thrombus into iliac vein in one patient, which required vascular surgical procedure. Minor complications included two cases of marginal flap necrosis, which settled with conservative treatment, and one case of implant loosening in an iliofemoral arthrodesis, which required revision surgery. Delayed wound healing was noted in patients who underwent preoperative radiotherapy. Functional outcome assessed by Enneking’s criteria 7 at the latest followup revealed excellent outcome in two patients, good outcome in five patients, and poor outcome in two patients. All our surviving patients are ambulant with orthotic support. The 5-year survival rate assessed by Kaplan Meier estimator was 63% and 10-year survival was 34%. None of the patients had local recurrence or radiation-induced secondary sarcomas.

## DISCUSSION

Ewing’s sarcoma of the pelvis is more aggressive than that at other sites and has an unfavorable prognosis. Delayed diagnosis is associated with larger tumors and micrometastasis, and results in poorer local and systemic control.^8^ Support services are important, with a requirement of large amount of blood products, specialized postrecovery care, and prolonged rehabilitation.^9^

With the advent of neoadjuvant chemotherapy and appropriate radiation therapy, and with accurate preoperative planning using computed tomography (CT), magnetic resonance imaging (MRI), and improvised surgical techniques, survival rates in patients with sarcoma have improved. Current principles of treatment involve a multimodal approach that involves local tumor control with attempts to preserve function, possible eradication of established micrometastases, and prevention of new systemic spread.^10^

Retrospective studies of the treatment of Ewing’s sarcoma lesions suggest that survival rates improve and that local recurrences decrease significantly when surgery is performed. In two studies from the Mayo Clinic, patients undergoing surgery with or without adjuvant chemotherapy had higher survival rates than those treated with radiotherapy or chemotherapy alone.^11^,^12^ Compared to those treated with radiotherapy and chemotherapy; there was a twofold increase in survival rates in patients undergoing surgery in addition to radiation and chemotherapy. In 1993, Frassica *et al*.^1^ from Mayo Clinic reported their experience with Ewing’s sarcoma of the pelvis. They found a 25% 5-year survival rate for 13 patients with localized disease treated with chemotherapy and radiation therapy alone, and a 75% 5-year survival rate in a group of 8 patients treated with chemotherapy, radiotherapy, and surgery. All our patients underwent preoperative chemotherapy with embolization if necessary and after surgery continued the chemotherapy. Radiotherapy was added if the surgical margins were contaminated as diagnosed on post-operative histopathological examination.

Wide resection is the crucial part of local treatment and has a much higher survival rate at 5 years (75%) compared with a rate of 25% in patients with marginal or intralesional resection.^13^ Lesions involving more than one pelvic area are usually larger than tumors confined to a single bone and it is difficult to achieve wide resection margins especially when the sacrum is involved. The tumor volume has been documented as a poor prognostic factor.^14^ Hence the decision of surgery is one that must be made on an individual basis. The functional and psychological outcome of the surgery must be weighed against its potential benefit.

### Classification of pelvic resections

Internal hemipelvectomy involves the resection of the entire hemipelvis or of a portion of the hemipelvis (innominate bone) with preservation of the ipsilateral extremity. The classification of internal hemipelvectomies [Figure 1] is based on the resected region of the innominate bone from posterior to anterior.^15^,^16^ Type I is the resection of the ilium, Type II involves the resection of periacetabular region, and Type III is resecting the ischiopubic region. Type IV is the excision of the total sacral ala, that may also be called an extended Type I or as a Type IV resection. In Types I-III, “A” indicates a more aggressive resection [Figure 1]. Reconstruction may be done by prosthetic means, biological means, or combination of endoprosthesis and bone grafts. The reconstructive options for the functional defect that follows internal hemipelvectomy or continuity resections are many. The advantages and disadvantages of any reconstructive procedure are well known.^4^ ·Arthrodesis or flail hip provides a better option than other methods in view of long-term functional rehabilitation.^17^ In our series since the patients were young and resection more often than not ended with little options for stable prosthetic fixation, no attempts at complicated reconstructive procedures were made. Fusion was attempted but could not be achieved in any of the cases even at the end of 3 years. The idea of reconstruction using various implants and fibular strut graft is to achieve some temporary stability in immediate postoperative period. Later as the fibrous tissue forms in these gaps, which we attempted to augment using meshes, these patients end up having some form of pseudoarthrosis with reasonably painless mobility. We believe that pseudoarthrodesis after attempted arthrodesis and patients with no reconstruction fared functionally well with less morbidity due to good tissue fibrosis and assistive orthotic devices providing the necessary stability and compensation for shortening.^18^ Our results also suggest a poor prognosis for those patients with tumors located in two adjoining pelvic bones (ilium, ischium, pubis, and sacrum). Postoperative radiotherapy has been shown to improve local control after an incomplete resection and in patients with a poor histological response.^19^

Long-term survivors of bone tumors during childhood or adolescence are also at high risk for secondary malignancies. The risk of subsequent sarcoma is particularly high in the irradiated bone of survivors of Ewing’s sarcoma.^19^,^20^ Some authors suggest the combination of chemotherapy with alkylating agents and radiotherapy may have an enhancing effect on the induction of these secondary sarcomas, but other studies have suggested no correlation between chemotherapy and secondary sarcomas. To minimize this risk, the lowest effective radiation dose for eradication of tumor has been recommended.^21^ If the response to chemotherapy is significant or wide excision of the tumor is possible without contamination, radiotherapy can be avoided. Our study had no secondary cancers or treatment-associated sarcomas.

We support the view that most complications in the treatment of pelvic Ewing’s sarcoma are caused not by the type of reconstruction but by the type of resection and that the best predictors for survival include a localized lesion amenable to wide resection and when surgery is performed at a center with the access to excellent oncological, radio therapeutic, and radiological support. Closer the resection to the sacroiliac joint, more difficult is the reconstruction.

## CONCLUSION

Ewing’s sarcoma of the pelvis is a difficult disease to diagnose early. Adjuvant chemotherapy and radiation therapy have improved the survival rates for other primary bone tumors, but the prognosis of this disease still remains poor. Although our study was limited by the small number of surviving patients, the results suggest that surgery may improve the survival of patients with this tumor. More patients and longer followup are necessary for conclusive evidence regarding improved clinical outcome when surgical resection is used in the treatment of pelvic Ewing’s sarcoma.^21^
